# How does the COVID-19 pandemic impact on population mental health? A network analysis of COVID influences on depression, anxiety and traumatic stress in the UK population

**DOI:** 10.1017/S0033291721000635

**Published:** 2021-03-16

**Authors:** Orestis Zavlis, Sarah Butter, Kate Bennett, Todd K. Hartman, Philip Hyland, Liam Mason, Orla McBride, Jamie Murphy, Jilly Gibson-Miller, Liat Levita, Anton P. Martinez, Mark Shevlin, Thomas V. A. Stocks, Frédérique Vallières, Richard P. Bentall

**Affiliations:** 1 University of Sheffield, Sheffield, UK; 2 University of Liverpool, Liverpool, UK; 3 Maynooth University, Maynooth, Republic of Ireland; 4 University College London, London, UK; 5 Ulster University, Northern Ireland; 6 Trinity College Dublin, Dublin, Republic of Ireland

## Abstract

**Background:**

The coronavirus disease 2019 (COVID-19) emergency has led to numerous attempts to assess the impact of the pandemic on population mental health. The findings indicate an increase in depression and anxiety but have been limited by the lack of specificity about which aspects of the pandemic (e.g. viral exposure or economic threats) have led to adverse mental health outcomes.

**Methods:**

Network analyses were conducted on data from wave 1 (*N* = 2025, recruited 23 March–28 March 2020) and wave 2 (*N* = 1406, recontacts 22 April–1 May 2020) of the COVID-19 Psychological Research Consortium Study, an online longitudinal survey of a representative sample of the UK adult population. Our models included depression (PHQ-9), generalized anxiety (GAD-7) and trauma symptoms (ITQ); and measures of COVID-specific anxiety, exposure to the virus in self and close others, as well as economic loss due to the pandemic.

**Results:**

A mixed graphical model at wave 1 identified a potential pathway from economic adversity to anxiety symptoms via COVID-specific anxiety. There was no association between viral exposure and symptoms. Ising network models using clinical cut-offs for symptom scores at each wave yielded similar findings, with the exception of a modest effect of viral exposure on trauma symptoms at wave 1 only. Anxiety and depression symptoms formed separate clusters at wave 1 but not wave 2.

**Conclusions:**

The psychological impact of the pandemic evolved in the early phase of lockdown. COVID-related anxiety may represent the mechanism through which economic consequences of the pandemic are associated with psychiatric symptoms.

## Introduction

From its beginning, it was recognized that the coronavirus disease 2019 (COVID-19) pandemic would likely create a burden on mental ill-health in the general population (Holmes et al., 2020). However, pandemics are multifaceted phenomena, exposing people not only to the risk of illness, but also to the social restrictions and potential economic hardships that are necessitated by efforts to control the spread of the virus. To mitigate the mental health effects of the pandemic, therefore, it is necessary to understand not only the magnitude of the overall burden, but also which facets make the greatest contribution to it. In this paper, we apply a recently developed analytical approach, network analysis, to address this question using data collected from the adult population during the early imposition of severe restrictions (lockdown) in the UK, revealing that economic factors are particularly impactful on the mental health of this cohort.

Prior to this study, numerous attempts have been made to determine the scale of the pandemic's impact on mental health, mostly by employing online survey methods necessitated by restrictions. The Wellcome Trust established the COVIDMINDS Network (https://www.covidminds.org/) to catalogue these projects and, at the time of writing, more than 100 longitudinal studies are registered there – although commentators have noted that many have methodological limitations, especially in sampling (Pierce et al., 2020b). In the UK, quality cross-sectional studies with representative samples early in the pandemic have reported elevated levels of anxiety and depression compared to previous reported prevalence rates (e.g. Pieh et al., 2020; Shevlin et al., 2020), a finding supported by an online follow-up of the UK Household Longitudinal Study, which showed an increase in common psychiatric disorders compared to interviewer-obtained scores in previous waves (Pierce et al., 2020a). Meta-analyses of the international literature also reported increases in population mental ill-health (Cooke, Eirich, Racine, & Madigan, 2020; Salari et al., 2020). A consistent finding has been that psychiatric disorders were most prevalent in young adults, women, and adults with young children – although whether this reflects the specific impact of the pandemic on them remains unclear as these groups were already known to be especially vulnerable to psychological disorders (Baxter, Scott, Vos, & Whiteford, 2013; Henderson et al., 1998; Ritesh, Stevens, Havinder, de Vogli, & Halfron, 2007; Scott et al., 2008; Skipstein, Janson, Kjeldsen, Nilsen, & Matheiesen, 2012). A further concern is that online methods may elicit more reporting of psychological distress, making comparisons with pre-pandemic data obtained by interviews problematic (Sagar, Chawla, & Sen, 2020).

### Network theory

Confidence about the deleterious mental health effects of the pandemic could be increased by showing that symptoms vary according to exposure to specific facets of the pandemic. The network approach provides a methodology that allows this goal to be achieved. This approach emerged in response to concerns about the limitations of the latent variable approach to psychopathology, which assumes that psychiatric syndromes occur because the component symptoms share latent common causes (Borsboom & Cramer, 2013). In contrast, network theorists hypothesize that mental disorders arise out of complex causal relationships between symptoms (McNally, 2016). For example, insomnia, fatigue and irritability are not conceived as products of a latent depression disease process but as covarying because they affect each other, e.g., insomnia leading to fatigue leading in turn to irritability. Psychiatric disorders are therefore conceived as emergent phenomena, in which the covariation between symptoms evolves over time, leading to an endpoint in which recognizable syndromes are evident. Psychiatric comorbidity, which is very common between depression and anxiety (Lamers et al., 2011), is difficult to explain within the latent variable approach which assumes that these disorders are independent phenomena (Aragona, 2009; Maj, 2005); but is easily accommodated within network models, which assume that experiencing the symptoms of one disorder, for example, low self-esteem in depression, can increase the likelihood of a symptom of another disorder, for example fear of the future in anxiety.

The novel analytical tools which have been inspired by network theory allow mental disorders to be represented graphically, as networks with ‘nodes’ representing symptoms and ‘edges’ representing inferred statistical associations between them. ‘Centrality indices’ can be computed to infer the importance of each node within the network, in terms of strength of connections to other nodes. The expectation is that this approach will lead to the identification of symptoms which are particularly important in the genesis of psychological disturbance or which ‘bridge the gap’ between different syndromes, thereby leading to comorbidity between disorders (Beard et al., 2016; McNally, 2016).

Until recently, these tools have been largely used inductively, to make data-driven, hypothesis-generating inferences about possible causal relations between symptoms. However, recent studies have sought to use these methods in a hypothesis-driven manner. Such studies, for instance, have included trauma-related variables in psychopathology networks (to infer pathways from environmental adversity to severe mental illness; Isvoranu et al., 2017); used measures of neighbourhood deprivation and trust (to show how harsh living environments may lead to paranoid symptoms; McElroy et al., 2019); and have included theoretically relevant variables in psychopathology networks to adjudicate between different theories (De Beurs et al., 2019).

### Aims and objectives of the study

Our overall aim is to understand how specific facets of the current pandemic are associated with psychopathology symptoms. Our measures were chosen in a theory-driven manner and included symptom measures and measures representing several pandemic-specific variables: perceived risk of illness, perceived exposure to viral infection (either by self or a close other) and economic hardship. Our data were collected from two waves of a longitudinal survey project, the COVID-19 Psychological Research Consortium Study (McBride et al., 2020), which has already reported a conventional cross-sectional analysis of the prevalence of psychological disorders during the first week of lockdown in March 2020 (wave 1) (Shevlin et al., 2020).

We had two objectives. The first was to identify putative pathways between particular facets of the pandemic and particular symptoms. For this purpose, we used a mixed graphical model on the data from wave 1 and made tentative predictions about specific pathways of pandemic-action based on previous research. First of all, because infection and illness are likely to be traumatic experiences, and given that high rates of post-traumatic stress have been reported amongst survivors of previous pandemics (Xiao, Luo, & Xiao, 2020), it seemed likely that personal infection by COVID-19 would lead to post-traumatic symptoms. Second, because anxiety is usually associated with the anticipation of future threat during periods of uncertainty (Grupe & Nitschke, 2013), it seemed likely that perceived risk of future infection would be associated with anxiety symptoms. In this case, however, the effect could be bidirectional because anxiety may affect risk perception (Shiloh, Wade, Roberts, Alford, & Biesecker, 2013). Finally, as debt and economic hardship are associated with all common psychiatric disorders (Meltzer, Bebbington, Brugha, Farrell, & Jenkins, 2012; Richardson, Elliott, & Roberts, 2013), it seems likely that the degree of economic impact from the pandemic will be associated with higher levels of both anxiety and depressive symptoms.

Our second objective was to test how the structure of psychiatric symptoms and their relationship with features of the pandemic changed between the first wave 1 of the survey, in late March, and wave 2 approximately one month later. For this purpose, we used the Ising model which allows for comparisons between networks. Because this model only accommodates binary data, we chose internationally recognized clinical cut-offs for each of the symptoms. Hence, in this analysis, we examine not only how various pandemic variables are associated with specific clinically significant symptoms, but also the extent to which these effects persist over time. The relations between the symptoms themselves, and whether they evolved over time (as hypothesized by network theorists; e.g. Borsboom & Cramer, 2013; McNally, 2016), were also of particular interest in this network. (It should be noted that when assessing network differences between two time points, it is assumed that the network structures represent stable states of the underlying psychopathology system at each time point.)

## Methods

### Sample

The COVID-19 Psychological Research Consortium (C19PRC) Study is an online longitudinal, multi-country survey. Participants in the UK strand, who were recruited by the survey company *Qualtrics*, were aged 18 or older, resident in the UK, and able to read and write in English. Ethical approval for the study was granted by the University of Sheffield (Ref no. 033759).

Quota sampling ensured that the sample was representative of the UK adult population in terms of age, sex and household income. Additionally, the baseline sample (wave 1 [W1]) was also representative of the UK population in relation to economic activity, ethnicity, and household composition (McBride et al., 2020). W1 (23–28 March 2020) recruited 2025 participants during the first week of UK lockdown. All W1 respondents were re-contacted and invited to participate in the wave 2 (W2), which was conducted during 22 April–1 May 2020. The W2 retention rate was 69.4% (*N* = 1406).

A detailed methodological account of the study, including fieldwork, sampling and design, quality control procedures, and sample characteristics at W1 and W2 can be found elsewhere (McBride et al., 2020). Briefly, at W1, the mean age of participants was 45.44 years (s.d. = 15.90; range 18–83 years); 51.7% were female (*n* *=* 1047), 48.0% (*n* = 972) were male and 0.3% (*n* = 6) checked the transgender, prefer not to say or other option. Most grew up in the UK (92.4%), were of white British/Irish ethnicity (85.5%), had a post-secondary level education (60.0%), gave a religious identification (62.1%) and were employed at the time of the W1 survey (48.8%). The sample was diverse in relation to urbanicity: city (24.6%), suburb (28.2%), town (30.6%) and rural (16.5%). Attrition between W1 and W2 resulted in the W2 sample having a higher proportion of males (52.7% *v.* 48.0% at W1), older people (59.8% aged 45 years or older *v.* 51.5%), higher earners (43.8% with a gross annual household income of ⩾£ 38 741 per annum *v.* 40.2%), people of white British/Irish ethnicity (88.1% *v.* 85.5%) and living outside cities (78.2% *v.* 75.4%), adults living in lone adult-only households (25.0% *v.* 22.4%), and individuals born in the UK (92.0% *v.* 90.6%). Meeting caseness criteria for depression, anxiety and PTSD were also bivariate predictors of attrition at W2 but, when entered into a model alongside the sociodemographic variables, they were not significant.

### Measures

Detailed information on our measures and coding strategy are outlined in online Supplementary Table S1. In brief, psychopathology outcomes were measured using validated scales for depression [Patient Health Questionnaire-9 (PHQ-9); Kroenke & Spitzer, 2002], generalized anxiety [Generalized Anxiety Disorder Scale (GAD-7); Spitzer, Kroenke, Williams, & Löwe, 2006] and traumatic stress [International Trauma Questionnaire (ITQ); Cloitre et al., 2018]. The ITQ was adapted specifically to measure traumatic stress related to COVID-19. Several predictor measures, formulated specifically for the purpose of the study, were also included: perceived COVID-19 infection of oneself or a close family member/friend; lost income as a result of the pandemic; worry about impact on household finances; specific anxiety about the pandemic and prospective perceived risk of being infected with COVID-19 within the next month. Most measures were identical at W1 and W2. However, to take into account the rapidly evolving situation in the UK at the beginning of the pandemic, the wording/measurement of the infection and lost income items changed slightly between W1 and W2 (see online Supplementary Table S1).

### Statistical analyses

R version 4.03 was used for all statistical analyses. The R code and the adjacency matrices of all networks to reproduce all results can be found in the online Supplementary Materials.

### Network estimation

All models were visualized as network graphs – wherein ‘nodes’ represent variables, and ‘edges’ represent the conditional dependencies between them (Epskamp & Fried, 2018), using the Fruchterman−Reingold algorithm from the qgraph R-package (Epskamp, Cramer, Waldorp, Schmittmann, & Borsboom, 2012).

*Objective 1:* To investigate how the six environmental predictors (i.e. the two economic variables, and four COVID-19 variables) were associated with symptoms at W1, all variables were included in a mixed graphical model (MGM) which can accommodate ordinal, binary, and continuous variables. The MGM of W1 was estimated on the full W1 sample (*N* = 2025) with the use of the R-package *mgm* (Haslbeck & Waldorp, 2020). The *mgm* estimation procedure employs a penalty approach that aims to control for potential spurious associations which would lead to false-positive findings, namely, the *least absolute shrinkage and selection operator* (LASSO) (Tibshirani, 1996). LASSO shrinks edge-weights, reducing smaller edges to zero. This procedure generates a variety of networks. To choose the most appropriate network, we used the Extended Bayesian Information Criterion (EBIC); (Foygel & Drton, 2010), setting its hyperparameter to default (*γ* = 0.5) to ensure a more conservative network estimation.

*Objective 2*: To be able to statistically compare the network structures of the W1 and W2 data, two Ising models for them were computed. The Ising model (see Van Borkulo et al., 2014) can be conceptualized as a probabilistic model, in which the joint distribution of a set of variables is represented by two sets of parameters: *threshold parameters* (i.e. the extent to which a given variable is being endorsed, ‘1’, or not, ‘0’) and *pairwise association parameters* (i.e. the edge-weights, which represent statistical associations between variables; in this case, logistic regression coefficients). Psychological researchers are typically interested in the latter parameters, which are indicative of conditional (*in*)dependencies among the variables. The more pairwise association parameters are estimated, however, the greater the likelihood of including spurious, false-positive edges (Epskamp & Fried, 2018). Thus, as with the *mgm* procedure, the eLASSO procedure, embedded within the IsingFit R-package, employs a penalty approach – also based on the EBIC (*γ* = 0.25) – by which only the most important associations between the variables remain in the network.

Listwise deletion is typically used to handle missing data in Ising models. After deleting a small number of cases with missing data at W2, complete data were available for 1386 individuals, and this same subsample was used to estimate the networks at both waves, enabling their comparison. Symptom scores were dichotomized using recommended clinical cut-offs (see online Supplementary Table S1).

### Network inference

Post-hoc analyses were conducted to draw inferences about both the local (specific features of the networks) and global network properties.

*Expected Influence* was computed for all networks. This centrality index (Opsahl, Agneessens, & Skvoretz, 2010) reflects the level of connectivity of a given node with the rest of the nodes in the network; it is the sum of all edge-weight values connected to a given node.

*Stability and Accuracy Analyses.* Psychometric investigations have indicated that centrality statistics are often unreliably estimated (Epskamp, Borsboom, & Fried, 2017). Therefore, post-hoc stability analyses for both centrality and edge-weight parameters were conducted using the *bootnet* R-package (see online Supplementary Material).

*Predictability Estimates* were derived for all variables in the W1 mgm-network, which were visually represented as pie charts around each of the nodes. These estimates quantify the extent to which nodes are predicted by other nodes in the network – akin to *R*^2^ in regression (Haslbeck & Fried, 2017).

*Network Comparison.* The two Ising network models (W1 and W2) were compared, using the network comparison test (NCT) from the R package ‘NetworkComparisonTest’ (Van Borkulo, Epskamp, & Milner, 2016). NCT assesses for differences between the networks in: (i) *global strength* (summed edge-weights of the networks), and (ii) *structural invariance* (statistically significant changes in relations between variables). For the latter, the analysis determines the largest individual differences in edge-weights between the two networks. A thousand random permutations were employed for the network comparison procedure.

*Community detection.* ‘Walktrap’ is an algorithm designed to detect clusters or ‘communities’ within a network (Pons & Latapy, 2005). The modularity ratio (also known as the Q-index) can be utilized to evaluate the goodness-of-fit of the communities. Modularity ranges from 0 to 1 with higher values indicating a greater likelihood of non-random communities (Newman & Girvan, 2004).

## Results

Descriptive statistics for the psychopathology and environmental variables at W1 are presented in Table 1.

**Table 1.** Psychopathology and environmental variable descriptive statistics at W1 (*N* = 2025)

Node	Label	*N* (%)				
		Not at all	A little bit	Moderately	Quite a bit	Extremely
ITQ1	Dreams	1339 (66.1%)	252 (12.4%)	240 (11.9)	142 (7.0)	52 (2.6)
ITQ2	Flashbacks	1382 (68.2)	232 (11.5)	229 (11.3)	130 (6.4)	52 (2.6)
ITQ3	Internal-Avoidance	1332 (65.8)	266 (13.1)	246 (12.1)	128 (6.3)	53 (2.6)
ITQ4	External-Avoidance	1312 (64.8)	253 (12.5)	258 (12.7)	149 (7.4)	53 (2.6)
ITQ5	On guard	923 (45.6)	426 (21.0)	336 (16.6)	240 (11.9)	100 (4.9)
ITQ6	Startled	1239 (61.2)	283 (14.0)	252 (12.4)	172 (8.5)	79 (3.9)
		Not at all	Several days	More than half the days	Nearly every day	
PHQ1	Interest	1124 (55.5)	498 (24.6)	282 (13.9)	121 (6.0)	
PHQ2	Depressed	1054 (52.0)	593 (29.3)	239 (11.8)	139 (6.9)	
PHQ3	Sleep	986 (48.7)	554 (27.4)	286 (14.1)	199 (9.8)	
PHQ4	Energy	984 (48.6)	595 (29.4)	252 (12.4)	194 (9.6)	
PHQ5	Eating	1320 (65.2)	372 (18.4)	214 (10.6)	119 (5.9)	
PHQ6	Self-image	1382 (68.2)	324 (16.0)	206 (10.2)	113 (5.6)	
PHQ7	Concentration	1299 (64.1)	410 (20.2)	206 (10.2)	110 (5.4)	
PHQ8	Restlessness	1670 (82.5)	178 (8.8)	126 (6.2)	51 (2.5)	
PHQ9	Suicidal ideation	1652 (81.6)	198 (9.8)	117 (5.8)	58 (2.9)	
GAD1	Nervous	898 (44.3)	669 (33.0)	268 (13.2)	190 (9.4)	
GAD2	Worrying	1105 (54.6)	516 (25.5)	242 (12.0)	162 (8.0)	
GAD3	Generalized worrying	1031 (50.9)	565 (27.9)	255 (12.6)	174 (8.6)	
GAD4	Relaxing	1043 (51.5)	575 (28.4)	249 (12.3)	158 (7.8)	
GAD5	Restlessness	1372 (67.8)	384 (19.0)	176 (8.7)	93 (4.6)	
GAD6	Irritability	1095 (54.1)	530 (26.2)	256 (12.6)	144 (7.1)	
GAD7	Negative future	1054 (52.0)	558 (27.6)	248 (12.2)	165 (8.1)	
		Yes			No	
EC.I^a^	Lost income	648 (32.0)			1377 (68.0)	
C.Self^a^	Perceived self-infection	48 (2.3)			1977 (97.7)	
C.Close^a^	Perceived infection in close family member/friend	112 (5.5)			1913 (94.5)	
		M (s.d.)					
EC.W	Economic worry	5.67 (2.85)					
C.Risk	Perceived COVID-19 risk	48.01 (26.13)					
C.Anx	COVD-19 anxiety	67.72 (24.60)					

a Items are measured differently at W2, see online Supplementary Table S1 for details.

### Objective 1: pathways from pandemic variables to symptoms at W1

**Fig. 1.**
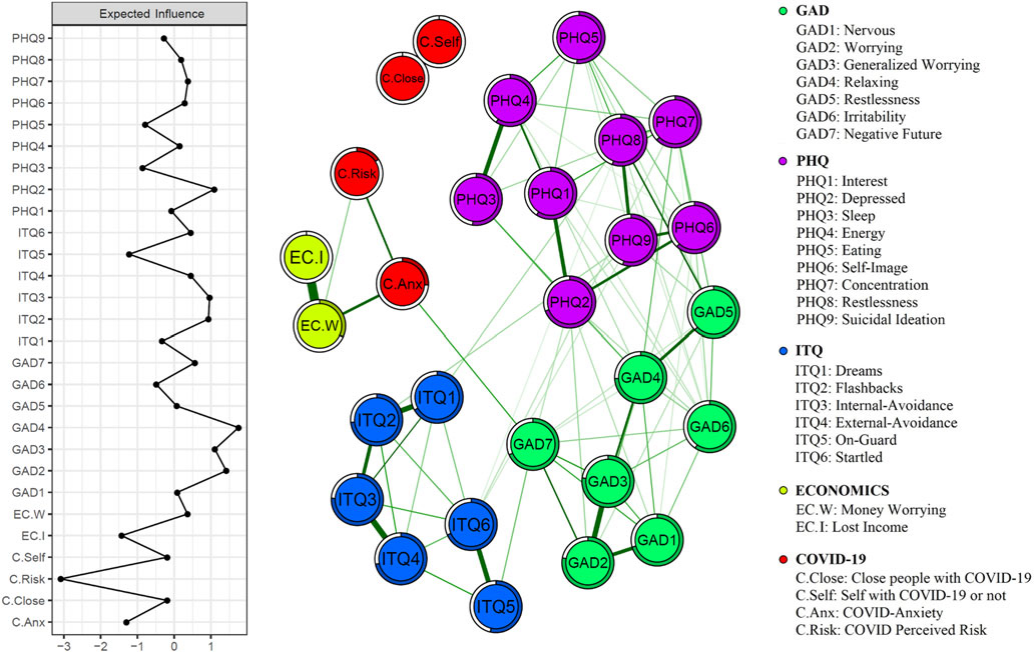
Mixed graphical model of anxiety, depression and traumatic stress symptoms in the UK population during the first week of lockdown. Expected influence statistics are shown in the panel on the left and the predictability of each node by other nodes is indicated by the circles surrounding each node.

The MGM network for the W1 data is presented in Fig. 1. Of 378 possible edges, only 84 were evident in the final graph. Amongst the pandemic variables, the strongest associations were found between the two economic variables (lost income and economic worry; *r* = 0.57); the two infection variables (perceived COVID-19 infection in self and others; *r* = 0.79); and economic worry and COVID anxiety (*r* = 0.24). The symptoms of each theoretical community (PHQ, GAD, and ITQ) clustered closer to one another, exhibiting greater within- compared to between-community connectivity. Bootstrapped confidence intervals validated the precision of the edge-weight parameters (see online Supplementary Fig. S1).

In the model, anxiety about the pandemic formed a bridge between perceived risk of infection and economic variables and the symptom variables, connecting specifically with GAD item 7, (‘feeling afraid as if something awful might happen’). In contrast, there was an absence of any connectivity between the two infection variables and mental health, and the trauma symptoms seem unconnected to any of the pandemic features.

*Centrality.* EI centrality statistics are also displayed in Fig. 1. Consistent with the above account, generalized anxiety (GAD) symptoms were the most interconnected aspects in the network as indicated by their high EI values. However, the EI statistics were relatively unstable (CS-coefficient = 0.13) and, as such, strong inferences should not be drawn from them.

*Predictability.* In line with the network theory of mental disorders, psychopathology symptoms were shown to have higher predictability values than the pandemic factors. The highest predictability values were exhibited by several GAD (e.g. GAD 2, GAD 3, and GAD 4, which scored 0.78, 0.77, 0.74, respectively) and ITQ symptoms (e.g. ITQ 2, ITQ 3, which scored 0.73 and 0.76, respectively). The pandemic factors had lower predictability values ranging from 0 (e.g. the two perceived-infection variables) to 0.32 (economic worry).

### Objective 2: comparison between networks at W1 and W2

**Fig. 2.**
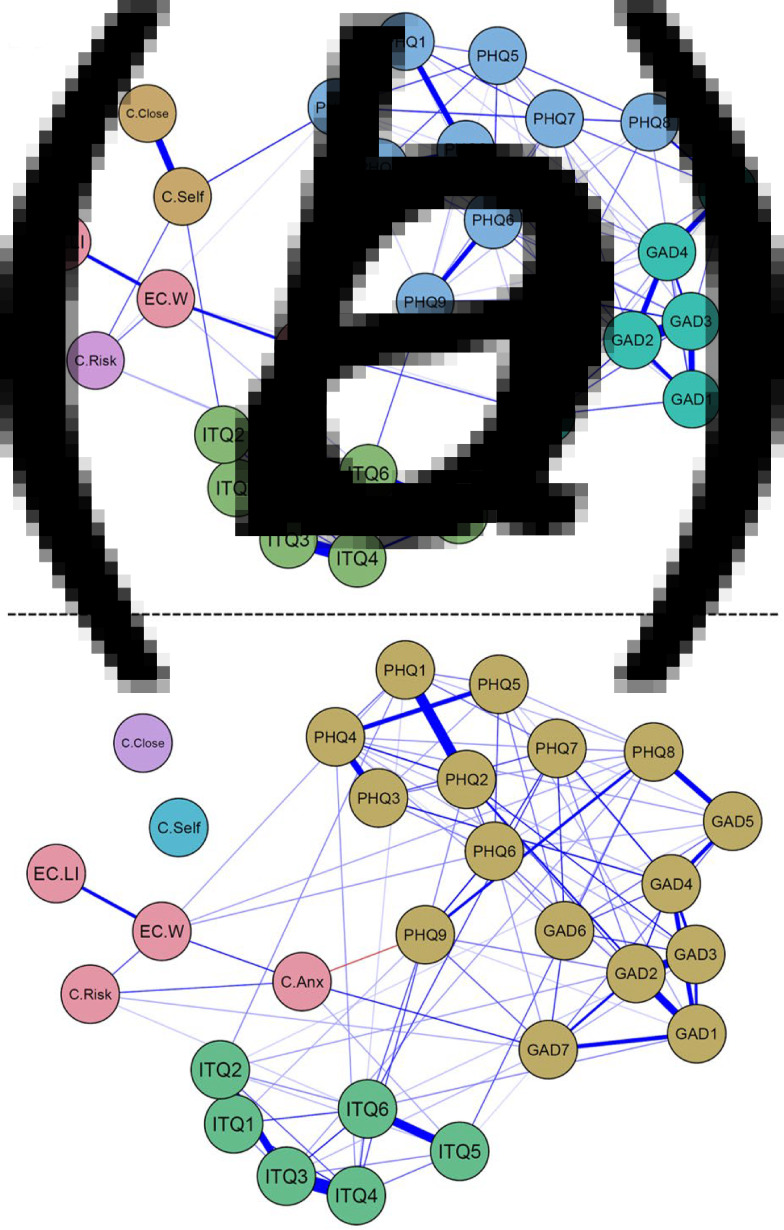
Ising network models for (*a*) wave 1 and (*b*) wave 2 data. Blue edges constitute positive associations, red edges constitute negative ones. Colour of node signifies their community, as determined by the walktrap algorithm.

*The Wave 1 Ising* model is shown in Fig. 2*a*. The Walktrap algorithm identified non-random clusters (modularity ratio = 0.49) of nodes in the network (indicated by node colour) corresponding to depression, anxiety and trauma symptoms. From 378 possible edges, 96 were retained in the final graph. Bootstrapped confidence intervals validated the accuracy of the edge-weight parameters (online Supplementary Fig. S2). In this network, the pandemic variables exhibited moderate partial correlations amongst one another, with notably strong associations existing between the two perceived infection variables (COVID infection of self and of others); and between the two economic variables (lost income and economic worry) as well as COVID anxiety. As in the MGM model, the GAD variables exhibited the highest EI values (see online Supplementary Fig. S10). In this case, EI values were determined to be very stable (*CS-Coefficient* = 0.75; see online Supplementary Materials for further details).

*The Wave 2 Ising model* is displayed in Fig. 2*b*; again, non-random clustering of nodes was indicated (modularity ratio = 0.34) but the community structure at W2 differed from W1, with depression and anxiety forming a single community. The W2 network exhibited much the same associations as the W1 model (e.g. 105 edges present from 378 possible ones) with some exceptions. Firstly, the two infection variables were not associated with other nodes. Additionally, a small, negative edge appeared between COVID-19 anxiety and PHQ 9 (suicidal thoughts). Bootstrapped confidence intervals validated the precision of the edge-weight parameters (see online Supplementary Fig. S3). Similar to W1, W2 EI estimates were highest for the GAD symptoms. Overall, EI values were stable at W2 (*CS-Coefficient* = 0.75; see online Supplementary Materials for further details).

*Network Comparison Test* (*NCT*). The two networks did not differ significantly in their Global Strength (GS) values (ΔGS = 2.28, *p* > 0.05). Thus, even though the wave 2 Ising model appears to be more strongly interconnected, this difference was not statistically significant. Contrasting this, however, the structural invariance test was significant (*M* = 1.62, *p* = 0.006) indicating that, although the two networks did not differ in terms of their global connectivity, they did differ in their largest edge weights.

Table 2 shows the list of edge weights that changed significantly between W1 and W2. Three such changes are noteworthy. Firstly, W1 relationships between infection of self and both PHQ4 (fatigue) and ITQ2 (flashbacks) were no longer evident at W2; secondly, perceived risk of infection and PHQ4 (fatigue) were no longer associated at W2; thirdly, an association between economic worry and PHQ6 (‘feeling bad about yourself, or that you are a failure, or have let yourself or your family down’) was evident only in the second wave. The changes in these associations between the two waves may possibly reflect the psychological progression of the pandemic.

**Table 2.** Edges with significantly different weights when the Ising models at waves 1 and 2 were compared (from NCT edge weight invariance test)

Edge pair	Significance	W1 edge weight	W2 edge weight
Changes in associations between pandemic variables			
COVID risk – COVID self	0.000	0.64515740	NA
COVID self – COVID close	0.000	1.61740220	NA
COVID anxiety – COVID risk	0.000	NA	0.7210435
Changes in association between pandemic variables and symptoms			
COVID self – PHQ4	0.000	0.94376077	NA
COVID self – ITQ2	0.001	0.73411176	NA
COVID risk – PHQ4	0.033	0.11981058	NA
Economic worry – PHQ6	0.017	NA	0.3978759
Changes in association between symptom variables			
PHQ2 – PHQ8	0.017	NA	0.4066655
GAD1 – PHQ2	0.019	NA	0.9604713
GAD3 – PHQ5	0.047	0.27500676	NA
GAD4 – GAD7	0.022	0.42123565	NA

## Discussion

The present investigation aimed to understand how various aspects of the pandemic were associated with symptoms of anxiety, depression and traumatic stress, and how these relations evolved over time.

### Associations between pandemic variables and mental health

The Network Theory of mental disorders emphasizes the interconnectivity of symptoms; hence the stronger edge weights between symptoms than between pandemic variables and symptoms was to be expected. Predictability indices were similarly higher for symptom nodes compared to pandemic nodes.

Overall, our models revealed that various aspects of the pandemic were differentially related to psychopathology symptoms. In particular, consistent across the two models and at both waves, lost income and economic worries were associated with specific anxiety about the pandemic, which in turn was associated with symptoms of generalized anxiety disorder via fears about the future (GAD-7). Perceived risk of infection was also associated with symptoms via anxiety about the pandemic (MGM and Ising model in wave 2). Although associations with depressive symptoms were less evident in the Ising models, fatigue (PHQ4) seemed to be associated with infection (wave 1) and economic worries (wave 2). Economic worries were also associated with feelings of failure (PHQ6) at wave 2, which is consistent with previous research showing that self-esteem mediates the relationship between financial hardship and psychological symptoms (Elahi et al., 2018).

The effects of perceived viral infection were more nuanced than expected. For instance, infection of self was associated with flashbacks (ITQ2), as hypothesized, but only in the wave 1 Ising model. Pandemic survivors who require intensive care are known to be at high risk of post-traumatic stress disorder (Xiao et al., 2020); however, the majority of infections reported in the present sample were likely mild. It is possible that the psychological effects of infection were more muted at wave 2 because people had become accustomed to the pandemic. Alternatively, this could be because, in the first week of lockdown, anyone reporting flashbacks was likely to have been currently infected; whereas, at wave 2, our assessment also captured those who had recovered from the coronavirus.

### Differences in network structure at W1 and W2

The Ising model allowed us to detect changes in network structure in the first month of lockdown. Notably, anxiety and depression symptoms formed separate communities in wave 1 but a single community in wave 2. It has long been known that comorbidity between these disorders is common (Lamers et al., 2011) and taxonomic studies of psychiatric disorders have converged on a model in which anxiety and depression form a single ‘internalizing’ dimension of psychopathology (Kessler et al., 2011; Krueger, 1999). Hence, a possible interpretation of the present findings is that separate syndromes of anxiety and depression coalesced into a single internalizing syndrome as the pandemic developed. The distinction of PTSD symptomology from the anxiety−depression cluster has been noted in other network studies, and could suggest that efforts to reduce PTSD symptomology may need to be directed separately from targeting anxiety−depression (Price, Legrand, Brier, & Herbert-Dufresne, 2019).

### Limitations

Although network theory implies a causal account of the evolution of psychiatric syndromes (Borsboom & Cramer, 2013; McNally, 2016), the analytical techniques employed here cannot reveal such causal dynamics. Nevertheless, in accordance with previous investigations, we have attempted to conduct our analyses in a theory-driven manner, making tentative predictions about how different aspects of the pandemic would affect the psychopathology network. Although our predictions were to a large extent supported, it should be noted that these effects could qualify as bidirectional given the undirected nature of the present networks.

Also, it is important to note that our Ising models were not used to compare symptoms and pandemic variables within individuals, but instead examined changes in the overall structures of the networks in a between-subjects manner. This comparison required us to remove from the W1 Ising model anyone who did not complete the survey at W2. Hence, changes observed between the W1 and W2 Ising networks are only applicable to this subsample, and may not be generalizable to the sample as a whole. This is particularly important given that this subsample comprised more psychologically healthy individuals.

Additionally, some minor changes in the wording of the items concerning COVID infection between the two waves means that aspects of the analyses that used these variables should be treated with caution. Objective measures of COVID infection and multi-item, psychometrically assessed scales measuring COVID-19 anxiety and financial worries could have made the findings more robust. Finally, our respondents were recruited by quota sampling and were not a true random probability sample. It should also be noted that the study was conducted in the UK during the earliest stages of the pandemic. As such, the results presented may be specific to UK general population and the context of the pandemic within the UK at that time (e.g. infection rates, government response).

### Future research

Of the environmental variables, economic worry had the highest predictability estimates in the MGM network, and the highest centrality estimates across waves 1 and 2 in the Ising models. However, given the overall predictability of economic worries was low−moderate, future studies should further explore what variables, over and above those currently included in the network, influence financial worry during the pandemic. In the MGM network, only one edge connected the environmental nodes to the mental health nodes (COVID-19 anxiety – Fear about the future) and directed networks could be used to further explore the directionality (uni- or bidirectional) of this pathway. It would also be informative to analyse cross-lagged relationships among the other variables in the network. The predictability estimates of the mental health variables suggest that they are highly interconnected, and future research might consider which symptoms could be targeted to maximize control of the network (Haslbeck & Fried, 2017).

### Implications

The currently observed associations between particular aspects of the pandemic and symptoms increase confidence that the pandemic created an increased burden of mental ill-health in the UK population during the early pandemic, as observed in more conventional analyses (Pieh et al., 2020; Pierce et al., 2020a; Shevlin et al., 2020). The importance of economic worries has obvious implications for the mental health of the nation. Hence, if government strategy during these difficult times is to maintain the psychological wellbeing of the population, continuing efforts to mitigate the economic consequences of the pandemic will be vital.

## Supplementary material

For supplementary material accompanying this paper visit http://dx.doi.org/10.1017/S0033291721000635. click here to view supplementary material
