# Supplementary material for: How does the COVID-19 pandemic impact on population mental health? A network analysis of COVID influences on depression, anxiety and traumatic stress in the UK population
Source: Psychol Med. 2021 Mar 16:1–9. doi: 10.1017/S0033291721000635 (PMC8010290; doi:10.1017/S0033291721000635)
Supplement: Supplementary file 1 [file S0033291721000635sup001.docx]

**Online supplementary material for Zavlis et al. (2021) ‘How Does The COVID-19 Pandemic Impact on Population Mental Health? A Network Analysis of COVID Influences On Depression, Anxiety and Traumatic Stress in the UK Population’**

| **Table S1.** Details of measures and coding strategy in the MGM and Ising networks | | | | |
| --- | --- | --- | --- | --- |
| Construct | Node name | Description | Measurement | Network coding |
| COVID-19 related PTSD | ITQ1 – ITQ6 | Post-traumatic stress disorder was assessed using the International Trauma Questionnaire (ITQ; Cloitre et al., 2018), a self-report measure of ICD-11 PTSD based on a total of six symptoms across the three symptom clusters of Re-experiencing, Avoidance, and Sense of Threat; each symptom cluster is comprised of 2 symptoms. Participants were asked to complete the ITQ as follows: “…in relation to your experience of the COVID-19 pandemic, please read each item carefully, then select one of the answers to indicate how much you have been bothered by that problem in the past month”. | All items are answered on a 5-point Likert scale, ranging from 0 (Not at all) to 4 (Extremely) with possible PTSD scores ranging from 0 to 24. A score of ≥ 2 (Moderately) is considered ‘endorsement’ of that symptom. The psychometric properties of the ITQ scores have been demonstrated in multiple general population (Ben‐Ezra et al., 2018; Cloitre et al., 2019) and clinical and high-risk samples (Hyland et al., 2017; Karatzias et al., 2016; Vallières et al., 2018) samples. | MGM network:   \| 0 = Not at all \| \| --- \| \| 1= A little bit \| \| 2= Moderately \| \| 3 = Quite a bit \| \| 4 = Extremely \|   Ising networks:  ≤ 1 = 0 (not endorsed)  ≥2= 1 (endorsed) |
| Depression | PHQ1 – PHQ9 | Depression was assessed with the Patient Health Questionnaire-9 (PHQ-9; Kroenke, Spitzer & Williams, 2001), a nine-item measure which corresponds to the DSM-IV Diagnostic Criterion A symptoms for major depressive disorder (American Psychiatric Association, 2000). Participants were asked how often, over the last two weeks, they had been bothered by each of the depressive symptoms. | Response options were “not at all”, “several days”, “more than half the days”, and “nearly every day”, scored as 0, 1, 2 and 3, respectively. PHQ-9 scores range from 0 to 27. Dichotomisation of the PHQ items for use in the Ising networks was based on the diagnostic algorithm for diagnosing depression using the PHQ-9 (“Instruction Manual”, n.d.). Psychometric properties of the PHQ-9 are well documented (see Kroenke, Spitzer, Williams & Löwe (2010) for an overview). | MGM network:   \| 0 = Not at all \| \| --- \| \| 1 = Several days \| \| 2 = More than half the days \| \| 3 =Nearly every day \|   Ising networks:  For PHQ1 – PHQ8:  ≤ 1 = 0 (not endorsed)  ≥2 = 1 (endorsed)  For PHQ9:  0 = 0 (not endorsed)  ≥1 = 1 (endorsed) |
| Anxiety | GAD1 -GAD7 | Experiences of generalized anxiety were assessed using the Generalized Anxiety Disorder Scale (GAD-7; Spitzer, Kroenke, Williams & Löwe, 2006). Respondents were asked to report how often in the past 2 weeks they were bothered by seven anxiety symptoms (e.g. trouble relaxing, becoming easily annoyed or irritable). | Response options were “not at all”, “several days”, “more than half the days”, and “nearly every day”, scored as 0, 1, 2 and 3, respectively. Dichotomisation of the GAD-7 items for use in the Ising networks was based on the framing of the DSM-5 criteria for Generalized Anxiety Disorder, i.e., “excessive anxiety and worry (apprehensive expectation), occurring more days than not…” (APA, 2013). The GAD-7 has demonstrated good reliability and construct validity, as evidenced by strong associations with other established measures of anxiety as well as diagnoses of GAD and its associations with depression, self-esteem, life satisfaction, and resilience (Löwe et al., 2008). | MGM network:   \| 0 = Not at all \| \| --- \| \| 1 = Several days \| \| 2 = More than half the days \| \| 3 =Nearly every day \|   Ising networks:  ≤ 1 = 0 (not endorsed)  ≥2 = 1 (endorsed) |
| COVID-19 anxiety | C.Anx | Respondents’ degree of specific anxiety about the COVID-19 pandemic was assessed using the item “How anxious are you about the coronavirus/COVID-19 pandemic?” | A single visual slider scale, ranging from 0 ‘not at all anxious’ on the left-hand side to 100 ‘extremely anxious’ on the right-hand side. | MGM network:  Continuous scale score  Ising networks:  The scores were recoded into quintiles, and the upper quintile was considered to be indicative of  ‘COVID-19 anxiety’. |
| COVID-19 perceived risk | C.Risk | Respondents estimated their perceived percentage risk of contracting COVID-19 within the next month using the item “What do you think is your personal percentage risk of being infected with the COVID-19 virus over the following time periods? (In the next month)” | A visual slider (ranging from 0% on the left-hand side to 100% on the right-hand side) | MGM network:  Continuous scale score  Ising networks:  The scores were recoded into quintiles, and the upper quintile was considered to be indicative of ‘High perceived COVID-19 risk’. |
| COVID-19 perceived infection status - self | C.Self | Perceived COVID-19 self-infection was measured differently at W1 and W2.  At W1 respondents were asked: “Have you been infected by the coronavirus COVID-19?” | At W1, 7 potential responses were given relating to potential infection status:  1=No. I have been tested for COVID-19 and the test was negative.  2=No. I do not have any symptoms of COVID-19.  3=I have a few symptoms of cold or flu but I do not think I am infected with the COVID-19 virus.  4=I have the symptoms of the COVID-19 virus and think I may have been infected.  5=I have been infected by the COVID-19 virus and this has been confirmed by a test.  6=I may have previously been infected with COVID-19 but this was not confirmed by a test and I have since recovered.  7=I was previously infected with COVID-19, this was confirmed by a test and I have now recovered. | W1 MGM and W1 Ising networks:  Responses 1,2,3,6 = No perceived infection  Responses 4,5, 7 = Perceived infection |
|  |  | At W2, respondents were asked if they had been tested for COVID-19 and if so, what was the outcome of that test.  Furthermore, respondents were asked if they were currently waiting to be tested for COVID-19.  Finally, respondents were presented with the following definition of ‘self-isolation’ and asked whether they were currently self-isolating, or had self-isolated in the past: ‘Self-isolation means that if you have COVID-19 symptoms, or if someone you live with has symptoms, you must not leave your home for between 7 - 14 days’ | Outcome of test responses: 1=Positive, 2=Negative  Waiting to be tested responses: 1=Yes, 2=No  Current/past self-isolation: 1 =Yes, 2=No | W2 Ising network:  Responses indicating a positive test result OR waiting to be tested OR current self-isolation OR previous self-isolation = Perceived infected  All other responses = No perceived infection |
| COVID-19 perceived infection – close family member/friend | C.Close | Perceived COVID-19 family member/friend infection was measured differently at W1 and W2.  At W1, respondents were asked: “Has someone close to you (a family member or friend) been infected by the coronavirus COVID-19?” | At W1, 4 potential responses were given relating to potential infection status of a close family member/friend:  1=No  2=Someone close to me has symptoms, but I am not sure if that person is infected  3=Someone close to me has symptoms, and I suspect that person has been infected  4=Someone who is close to me has had a COVID-19 virus infection confirmed by a doctor | W1 MGM and W1 Ising networks:  Responses 1 & 2 = No perceived infection  Responses 3 & 4= Perceived infection |
|  |  | At W2, respondents were firstly asked whether else from their household (other than themselves) been diagnosed with COVID-19 (confirmed by a test).  Additionally, they were also asked whether anyone from their extended family or network of friends had been diagnosed with COVID-19 (confirmed by a test)? | Close family member/friend infection responses: 1=Yes  2=No  3=Not applicable | W2 Ising network:  Responses indicating someone from their household OR someone from their extended family or friend network had been diagnosed = Perceived close other infection  All other responses = No perceived infection |
| Lost income | EC.I | Lost income as a result of the pandemic was measured differently at W1 and W2.  At W1, respondents were asked: ‘Some people have lost income because of the coronavirus COVID-19 pandemic, for example because they have not been able to work as much or because business contracts have been cancelled or delayed. Please indicate whether your household has been affected in this way.’ | At W1, 3 potential responses were given relating to lost income:  1=My household has lost income because of the coronavirus (COVID-19) pandemic  2=My household has not lost income because of the coronavirus (COVID-19) pandemic  3=I do not know whether my household has lost income because of the coronavirus (COVID-19) pandemic | W1 MGM and W1 Ising networks:  Response 1 = lost income  Responses 2 & 3 = no lost income |
|  |  | At W2, respondents were asked: ‘Please estimate the percentage change (either increase or decrease) in your monthly household income compared to the average monthly income before the COVID-19 pandemic’ | A visual slider scale centred at 0 and ranged from 100% (decrease) on the left-hand side to 100% (increase) on the right-hand side. | W2 Ising network:  Any negative value on the slider = lost income  Any positive value on the slider = no lost income |
| Economic worry | EC.W | Respondents were asked: ‘On balance, how much are you worried about the way that your household finances have been affected by the coronavirus COVID-19 pandemic SO FAR?’ | Scale ranging from ‘1 = Not at all worried’ to ‘10= Extremely worried’ | MGM network:  Continuous scale score 1-10  Ising networks:  The scores were recoded into quintiles, and the upper quintile was considered to be indicative of ‘Economic worry’. |

**Supplementary analyses**

For the following methodological procedures, the reader is referred to: Epskamp, Borsboom, & Fried (2018). The following analyses were conducted with the use of the R package ‘bootnet’ (Epskamp et al., 2018). For the visualization of the plots, the R package ‘ggplot2’ was used (Wickham, 2016).

***Accuracy of edge-weights***


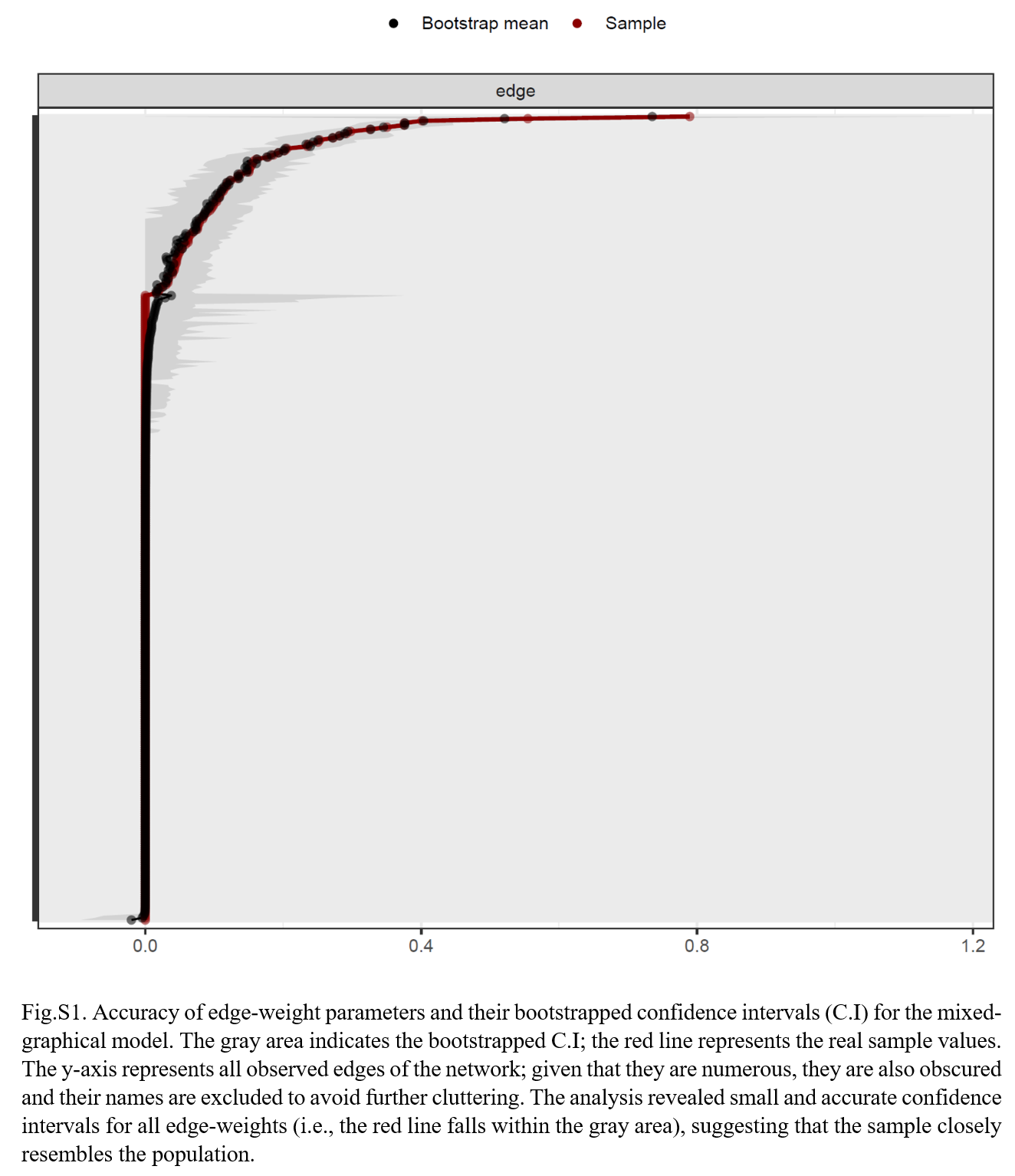
The following figure presents the results of the non-parametric bootstrapping procedure for the network’s edge-weight accuracy. The sample edge values (red line) fall within the bootstrapped confidence intervals (grey area), suggesting accurate edge-weight estimations.


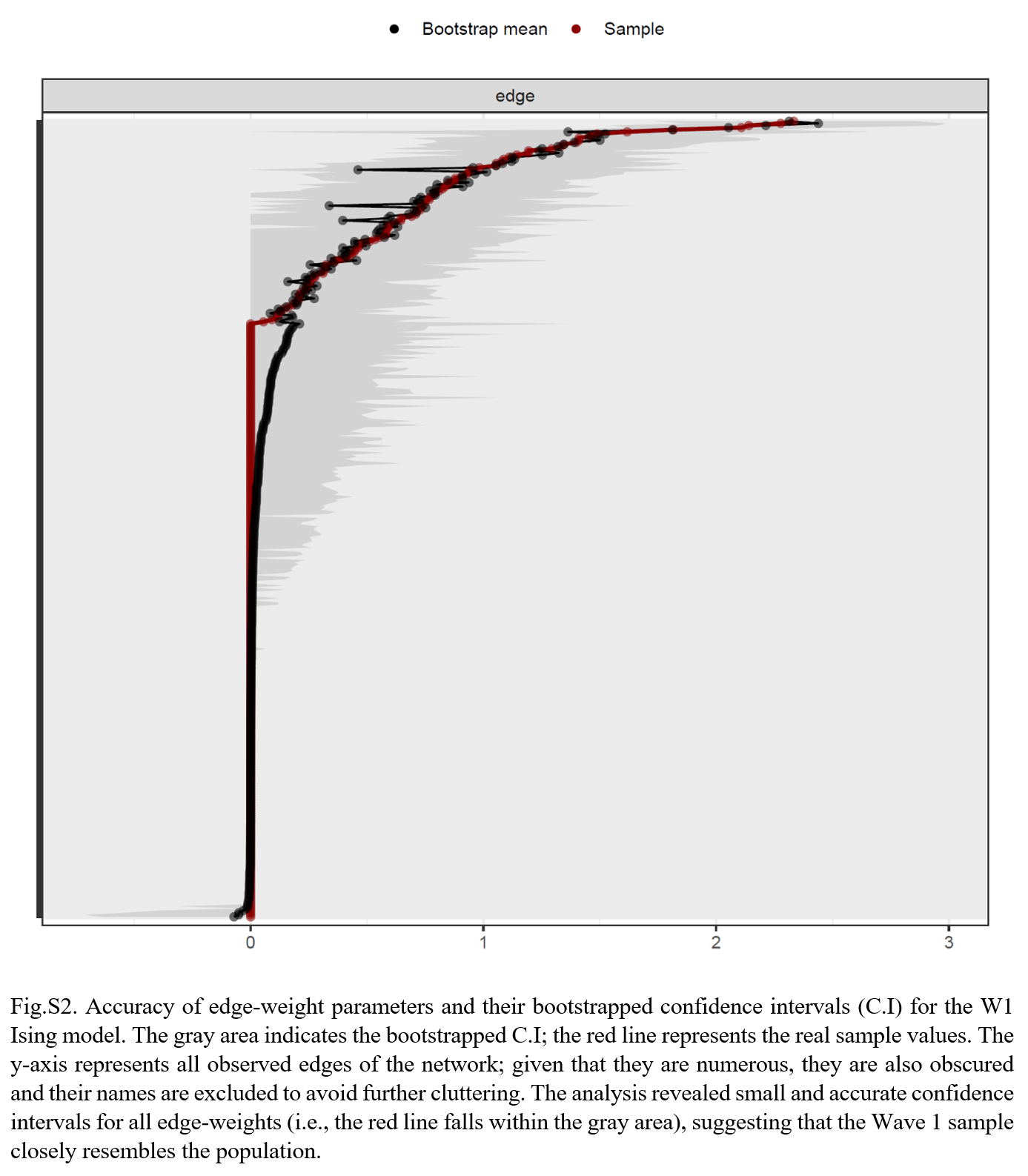


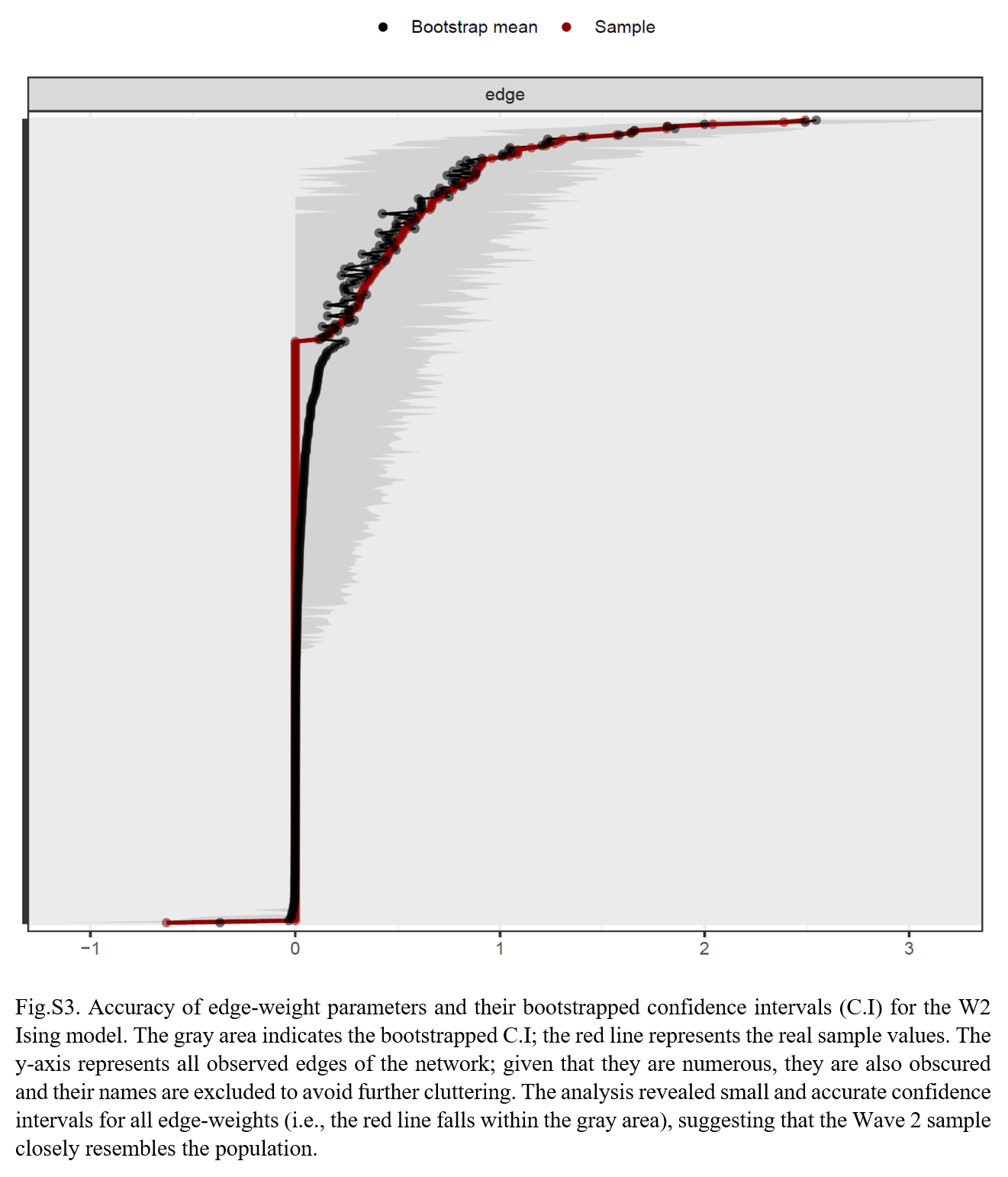


***Stability of Centrality Indices***

To assess the stability of centrality indices, the case-dropping subset bootstrap was employed. A more detailed account on this methodological approach can be found elsewhere (Epskamp, Borsboom, & Fried, 2018).


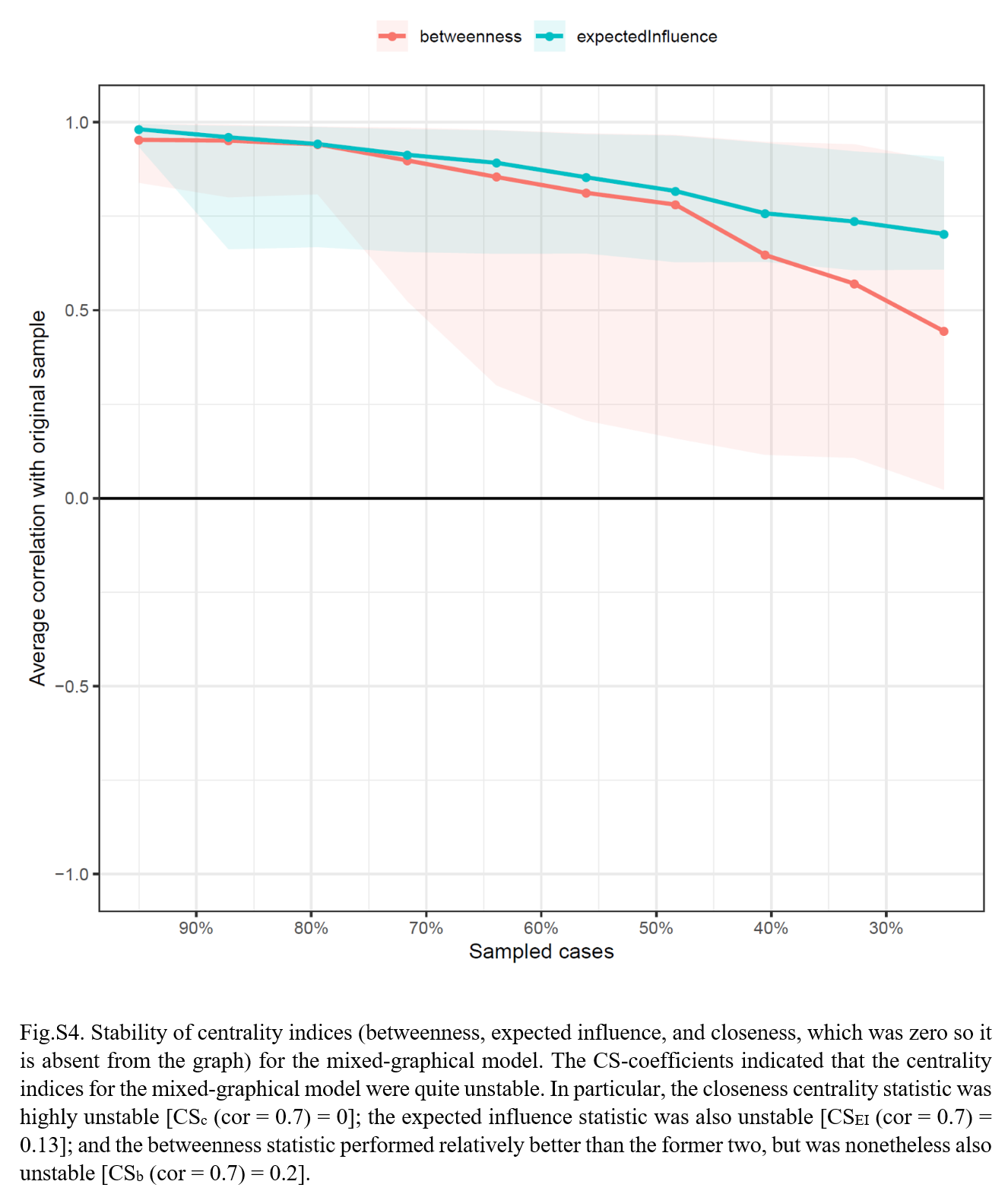


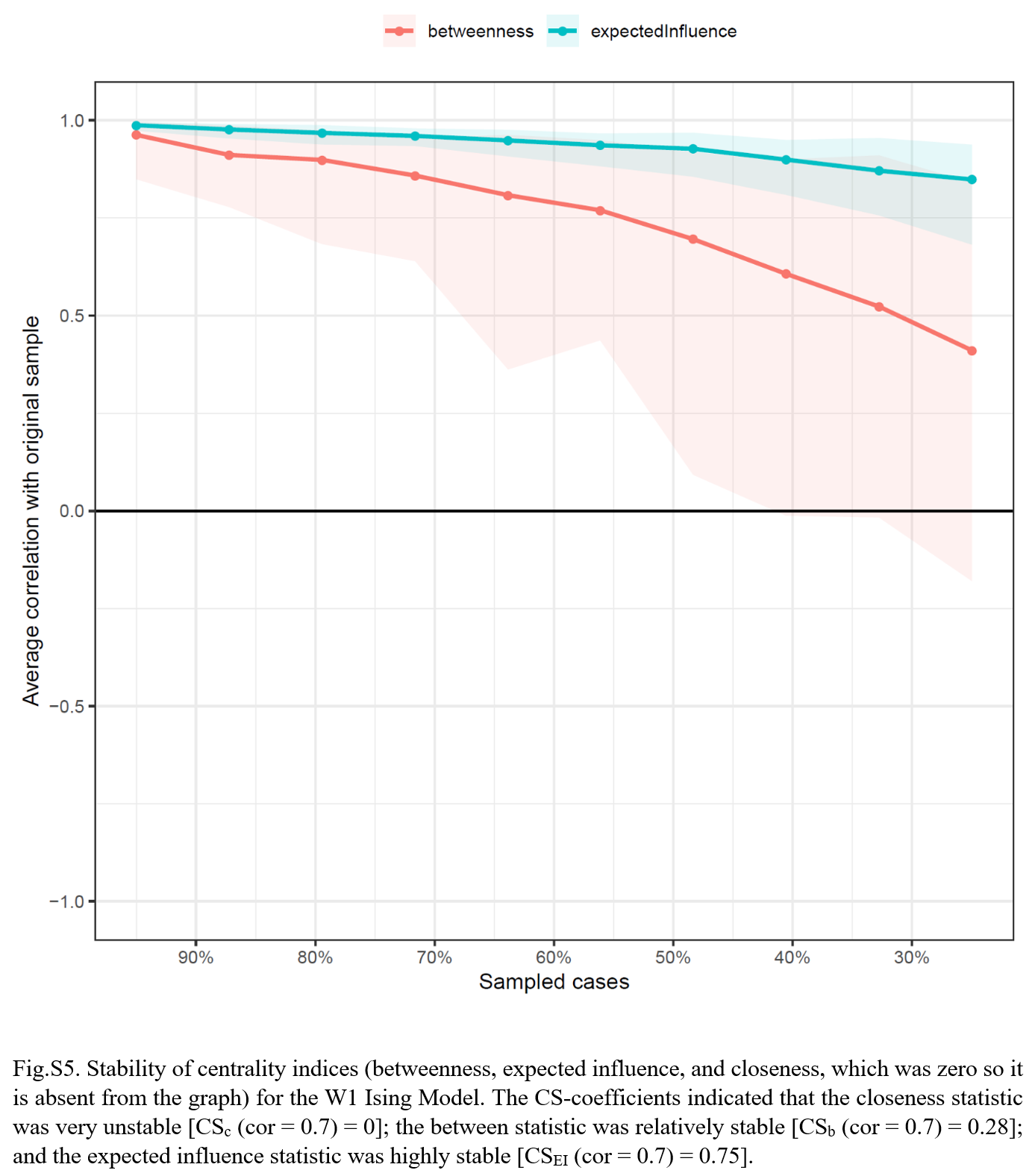


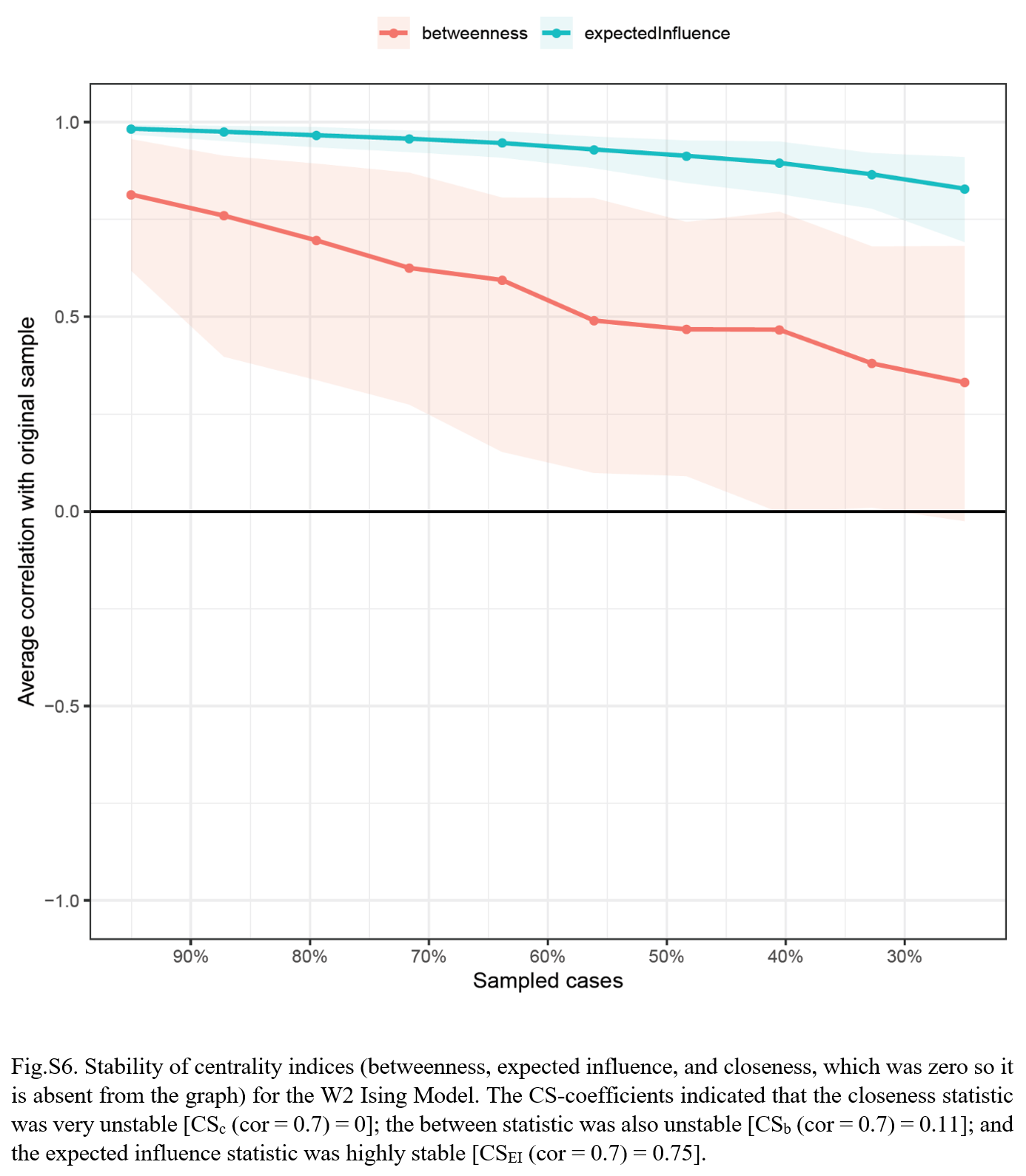


***Differences between edge-weights***

To assess whether significant differences between the edges-weight parameters exist, the non-parametric bootstrap procedure was employed, as described in Epskamp, Borsboom, & Fried (2018).


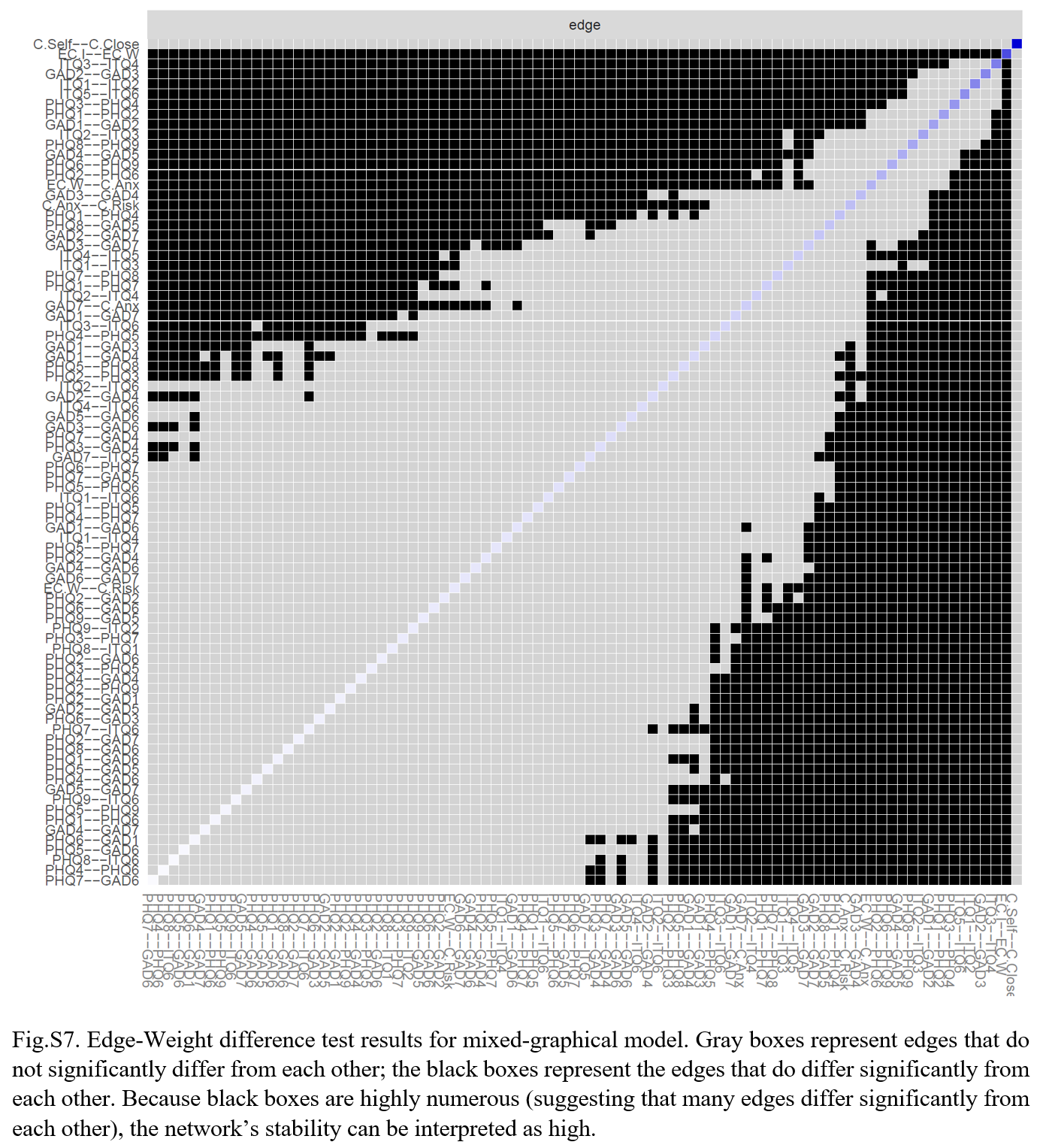


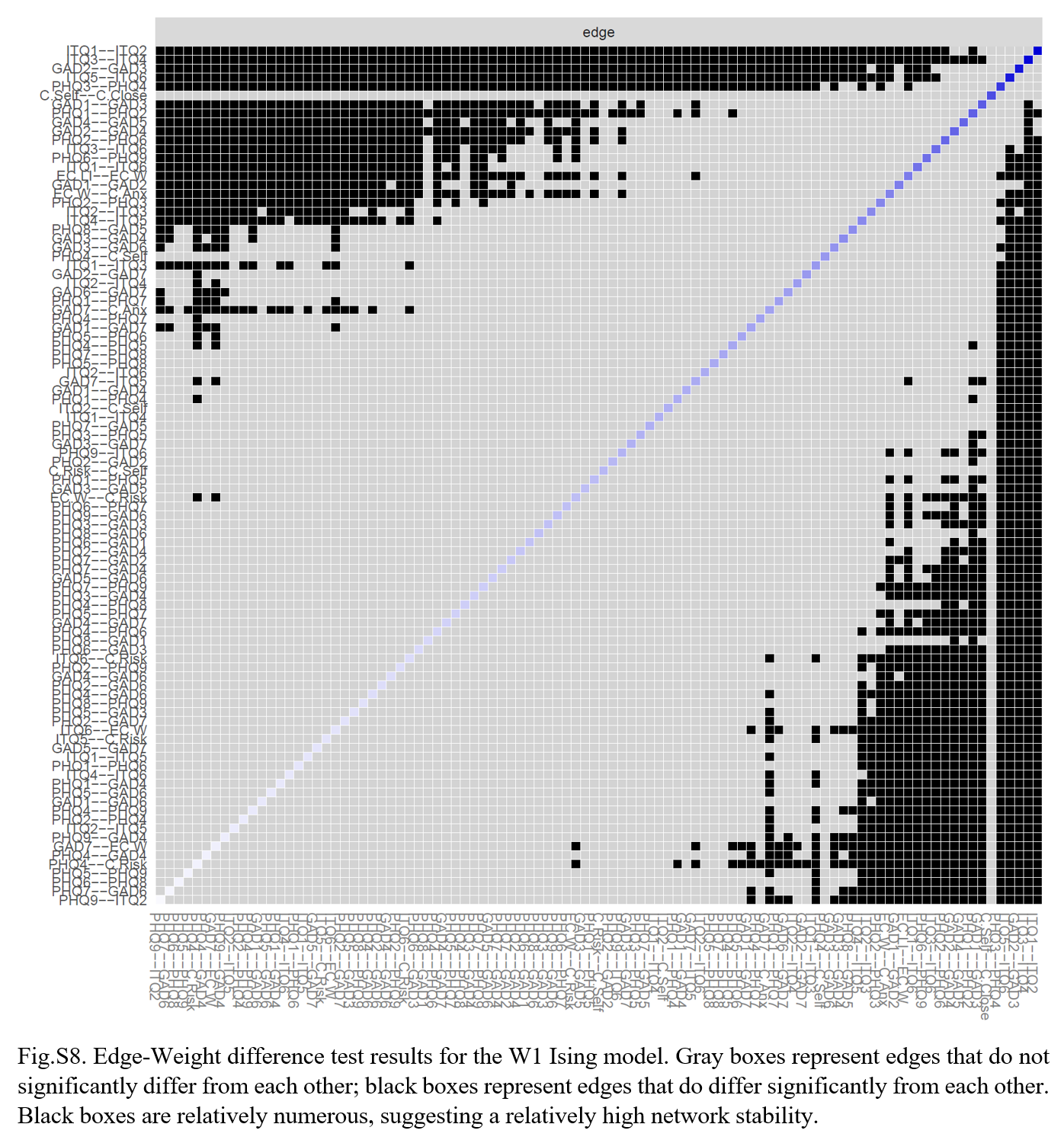


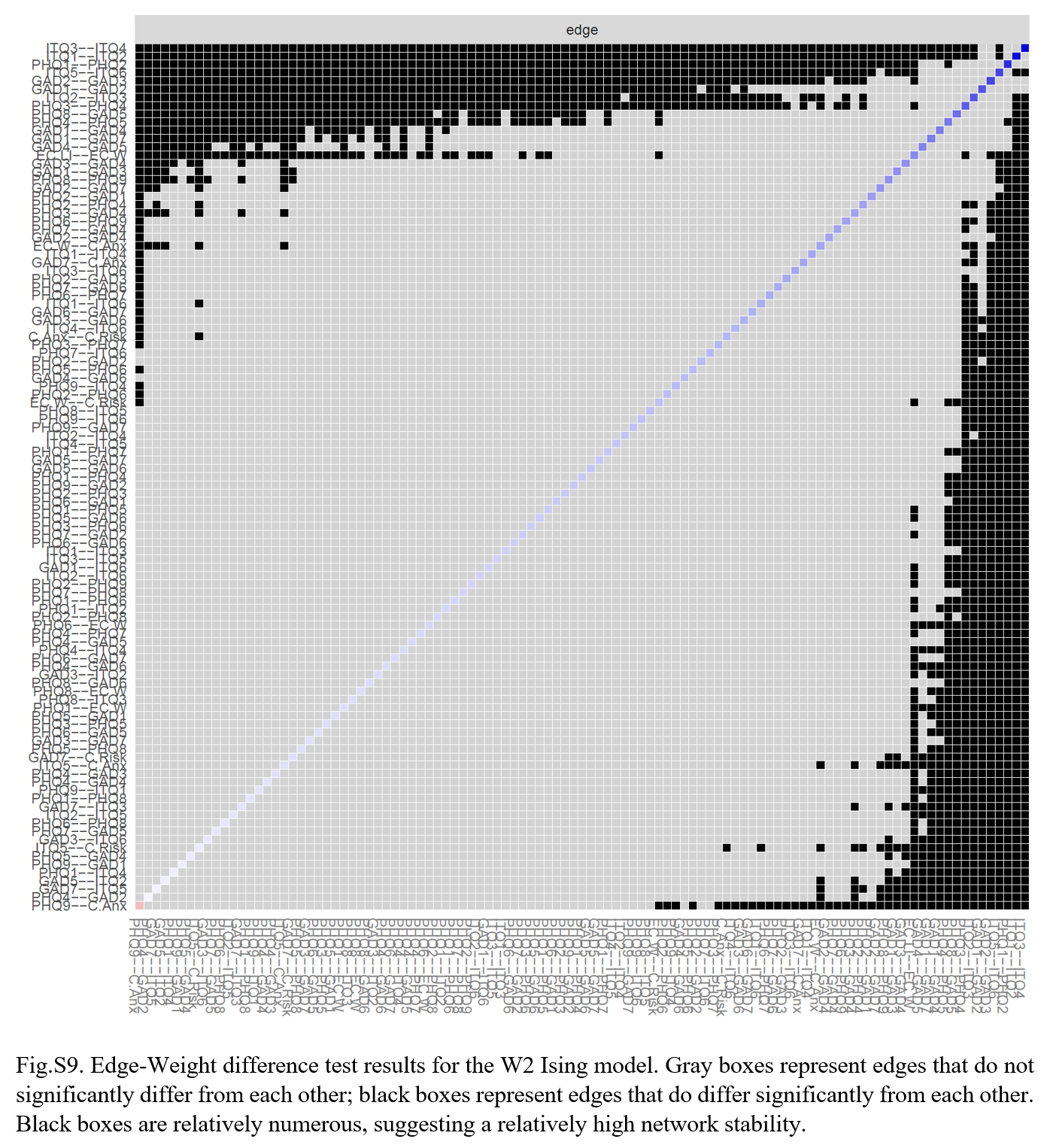


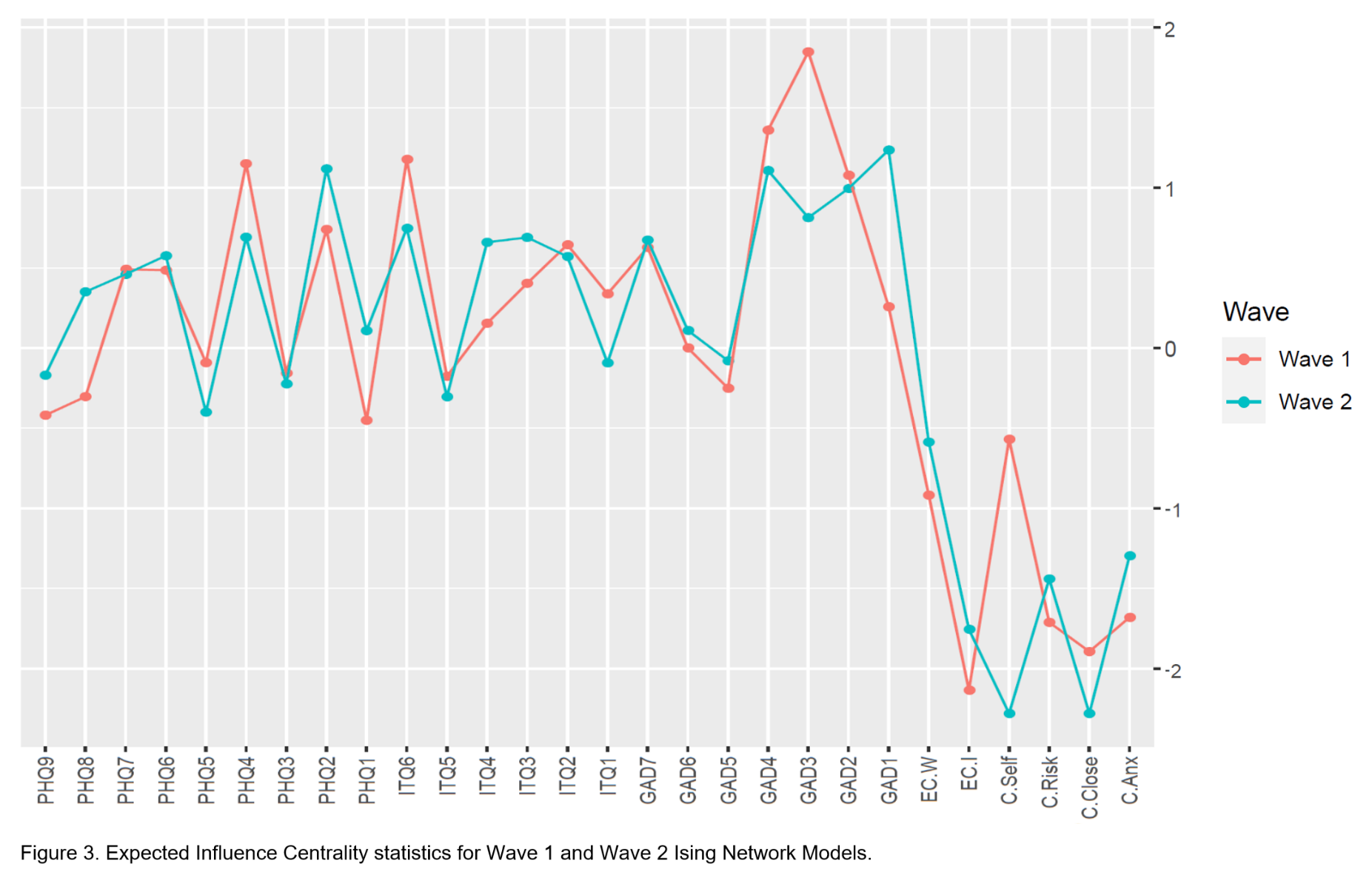


Fig S10. Expected Influence centrality statistics for Wave 1 and Wave 2 Ising network models

**References**

American Psychiatric Association. (2000). *Diagnostic and statistical manual of mental disorders: DSM-IV-TR*. Washington, DC: American Psychiatric Association.

American Psychiatric Association. (2013). *Diagnostic and statistical manual of mental disorders* (5^th^ ed.). Arlington, VA: American Psychiatric Publishing.

Ben‐Ezra, M., Karatzias, T., Hyland, P., Brewin, C. R., Cloitre, M., Bisson, J. I., ... & Shevlin, M. (2018). Posttraumatic stress disorder (PTSD) and complex PTSD (CPTSD) as per ICD‐11 proposals: A population study in Israel. *Depression and anxiety*, *35*(3), 264-274.

Cloitre, M., Hyland, P., Bisson, J. I., Brewin, C. R., Roberts, N. P., Karatzias, T., & Shevlin, M. (2019). ICD‐11 posttraumatic stress disorder and complex posttraumatic stress disorder in the United States: A population‐based study. *Journal of Traumatic Stress*, *32*(6), 833-842.

Cloitre, M., Shevlin, M., Brewin, C. R., Bisson, J. I., Roberts, N. P., Maercker, A., ... & Hyland, P. (2018). The International Trauma Questionnaire: development of a self‐report measure of ICD‐11 PTSD and complex PTSD. *Acta Psychiatrica Scandinavica*, *138*(6), 536-546.

Epskamp, S., Borsboom, D., & Fried, E. I. (2018). Estimating psychological networks and their accuracy: A tutorial paper. *Behavior Research Methods*, *50*(1), 195-212.

Hyland, P., Shevlin, M., Brewin, C. R., Cloitre, M., Downes, A. J., Jumbe, S., ... & Roberts, N. P. (2017). Validation of post‐traumatic stress disorder (PTSD) and complex PTSD using the International Trauma Questionnaire. *Acta Psychiatrica Scandinavica*, *136*(3), 313-322.

*Instruction manual: Instructions for Patient Health Questionnaire (PHQ) and GAD-7 measures.* (n.d.). PHQ Screeners. Retrieved from <https://www.phqscreeners.com/images/sites/g/files/g10016261/f/201412/instructions.pdf>

Karatzias, T., Shevlin, M., Fyvie, C., Hyland, P., Efthymiadou, E., Wilson, D., ... & Cloitre, M. (2016). An initial psychometric assessment of an ICD-11 based measure of PTSD and complex PTSD (ICD-TQ): Evidence of construct validity. *Journal of Anxiety Disorders*, *44*, 73-79.

Kroenke, K., Spitzer, R. L., & Williams, J. B. (2001). The PHQ‐9: Validity of a brief depression severity measure. *Journal of General Internal Medicine*, *16*(9), 606-613.

Kroenke, K., Spitzer, R. L., Williams, J. B., & Löwe, B. (2010). The patient health questionnaire somatic, anxiety, and depressive symptom scales: a systematic review. *General Hospital Psychiatry*, *32*(4), 345-359.

Löwe, B., Decker, O., Müller, S., Brähler, E., Schellberg, D., Herzog, W., & Herzberg, P. Y. (2008). Validation and standardization of the Generalized Anxiety Disorder Screener (GAD-7) in the general population. *Medical Care*, 266-274.

Spitzer, R. L., Kroenke, K., Williams, J. B., & Löwe, B. (2006). A brief measure for assessing generalized anxiety disorder: The GAD-7. *Archives of Internal Medicine*, *166*(10), 1092-1097.

Vallières, F., Ceannt, R., Daccache, F., Abou Daher, R., Sleiman, J., Gilmore, B., ... & Hyland, P. (2018). ICD‐11 PTSD and complex PTSD amongst Syrian refugees in Lebanon: the factor structure and the clinical utility of the International Trauma Questionnaire. *Acta Psychiatrica Scandinavica*, *138*(6), 547-557.

Wickham, H. (2016). *ggplot2: elegant graphics for data analysis*. Springer.
